# Critical fluctuations in a confined driven-dissipative quantum condensate

**DOI:** 10.1126/sciadv.adi6762

**Published:** 2024-03-22

**Authors:** Hassan Alnatah, Paolo Comaron, Shouvik Mukherjee, Jonathan Beaumariage, Loren N. Pfeiffer, Ken West, Kirk Baldwin, Marzena Szymańska, David W. Snoke

**Affiliations:** ^1^ Department of Physics, University of Pittsburgh, 3941 O’Hara Street, Pittsburgh, PA 15218, USA.; ^2^ Department of Physics and Astronomy, University College London, Gower Street, London, WC1E 6BT, UK.; ^3^ Joint Quantum Institute, University of Maryland and National Institute of Standards and Technology, College Park, MD 20742, USA.; ^4^ Department of Electrical Engineering, Princeton University, Princeton, NJ 08544, USA.

## Abstract

Phase fluctuations determine the low-energy properties of quantum condensates. However, at the condensation threshold, both density and phase fluctuations are relevant. While strong emphasis has been given to the investigation of phase fluctuations, which dominate the physics of the quantum system away from the critical point, number fluctuations have been much less explored even in thermal equilibrium. In this work, we report experimental observation and theoretical description of fluctuations in a circularly confined nonequilibrium Bose-Einstein condensate of polaritons near the condensation threshold. We observe critical fluctuations, which combine the number fluctuations of a single-mode condensate state and competition between different states. The latter is analogous to mode hopping in photon lasers. Our theoretical analysis indicates that this phenomenon is of a quantum character, while classical noise of the pump is not sufficient to explain the experiments. The manifestation of a critical quantum state competition unlocks possibilities for the study of condensate formation while linking to practical realizations in photonic lasers.

## Teaser

Evidence of mode competition due to quantum fluctuations is demonstrated in a non-equilibrium condensate near the critical point.

## INTRODUCTION

The experimental realization of Bose-Einstein condensates (BECs) enabled investigations of macroscopic quantum systems. One fundamental question relates to the role of fluctuations close to the critical point of a BEC transition. Critical quantum fluctuations arise from the uncertainty principle and play an essential role in a wide range of physical phenomena such as the structure of the universe, the Casimir effect, and transitions between two competing phases of matter (*1*–*8*). Understanding these fluctuations is important for statistical physics of phase transitions and quantum critical phenomena.

Up to now, the focus has largely been on phase fluctuations, which are fundamental for understanding the physics away from the critical point (*9*). Yet, at the threshold of condensation, density fluctuations play a crucial role, but they have been much less explored. In the context of equilibrium systems, number fluctuations have been characterized to some extent in dilute atomic gases (*10*–*12*) and photon in a dye (*13*, *14*) condensates. For the out-of-equilibrium systems, however, the physics of critical number fluctuations has not been explored yet. Here, the scenario is more complicated; in addition to the usual density fluctuations in a single lowest energy state, we can witness a competition between different modes in the condensate formation process, which is particularly pronounced at the vicinity of the critical point, where the condensate occupation is low.

Because of their strong nonlinear properties, microcavity exciton-polaritons (called here simply “polaritons”) are the perfect candidates for the investigation of critical density fluctuations in nonequilibrium quantum systems. They are quasiparticles arising from the strong coupling of cavity photons and excitonic transitions, typically in semiconductor quantum wells (QWs) placed at the antinodes of an optical cavity. Polaritons can be viewed as photons with nonlinear interactions or alternatively as excitons with very light effective mass (typically 10^−4^ times the mass of an electron in vacuum). As interacting bosons, polaritons demonstrate quantum phenomena such as Bose-Einstein condensation (*15*, *16*), superfluidity (*17*), and quantized vortices (*18*–*23*) at temperatures from tens of Kelvin up to room temperature.

In the present work, we undertake a detailed experimental and theoretical investigation of fluctuations near the condensate transition in a nonequilibrium system of nonresonantly pumped polaritons placed in an annular trap geometry. We explore theoretically the effect of quantum and classical noise on the system behavior and particularly on the mechanisms of the density fluctuations and mode competition. Apart from the fundamental interest, our results are also relevant for practical design of photonic lasers, where multimode switching—originating from their weakly-interacting nature—leads to “telegraphic noise” (*24*–*27*) instabilities.

## RESULTS

**Fig. 1.**
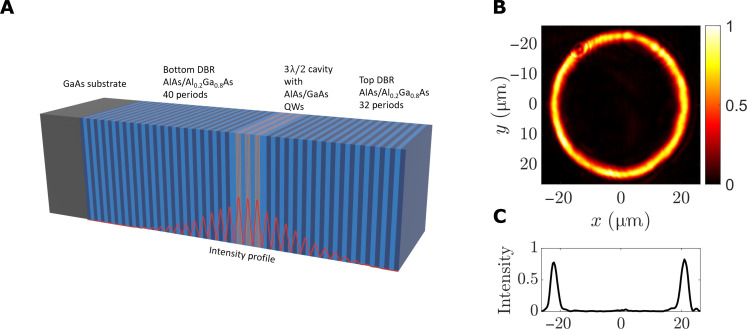
Optical trapping using an annular pump in a GaAs microcavity. (**A**) Microcavity structure used in the experiment. The dark and light blue layers represent the DBRs used to confine the light field. The yellow layers indicate the QWs, where bound excitons are formed, and the red lines indicate the intensity of the confined optical field. (**B**) Normalized real-space image of the excitation laser generated by a SLM. (**C**) A slice of the intensity of the excitation ring at the *y* = 0 line through the center of the excitation ring pattern shown in (B).

To investigate the fluctuations close to the critical point of condensation, we confined the polariton fluid in a circular trap using a spatial light modulator (SLM) to focus the pump laser in a ring, as shown in Fig. 1B. The pump laser acted as an incoherent source of excitons and polaritons because it had photon energy well above the exciton energy, so that many phonons had to be emitted before the hot carriers cooled and turned into excitons and polaritons. This incoherent pumping therefore had two effects: It created both the potential energy profile of the trap due to the repulsion of polaritons from the slow-moving excitons in the pump region, and it also acted as the source of the polaritons, as excitons converted into polaritons. As the pump power increased, the system went from noncondensed to a single-mode condensed regime, as reported in previous work. The large number of distributed Bragg reflector (DBR) periods gives the cavity a high *Q*-factor, resulting in a cavity lifetime of ∼135 ps and a polariton life time of ∼270 ps at resonance (see Materials and Methods for a detailed description) (*28*–*30*).

The sample was cooled in a continuous-flow cold-finger cryostat, which was held at a temperature of ∼5 K. To produce polaritons, the sample was excited nonresonantly with a continuous-wave laser, which was modulated by an optical chopper at 404 Hz with a duty cycle of 1.7% to prevent sample heating. The ring-shaped trap was generated by modulating the phase front of the laser using an SLM.

The diameter of the ring-shaped optical trap used in this manuscript was 45 μm, which is shown in Fig. 1B. The cavity detuning from the exciton resonance was δ = −10.1 meV, corresponding to an exciton fraction ∣*X*∣^2^ = 0.3 for the lower polariton. The photoluminescence (PL) was collected using a microscope objective with a numerical aperture of 0.75 and was imaged onto the entrance slit of a spectrometer. The image was then sent through the spectrometer to a charge-coupled device (CCD) for time-integrated imaging.

Polariton condensates allow the study of a range of behavior as the detuning, and consequently the photon fraction of the polaritons, is changed. In the limit of high exciton fraction, the modes are closely spaced together in a near continuum, and the interactions between the particles are strong, so that the dynamics are those of a close to equilibrium BEC (*31*, *32*). As the exciton fraction is decreased, the interactions become weaker and their mass lighter, so that they approach behavior that is more like a standard laser. The experiments we have performed here are at a middle ground; the polaritons are in the strong coupling limit, and travel tens of microns away from the pump region but have resolvable transverse modes similar to those of a vertical cavity surface-emitting laser (VCSEL).

**Fig. 2.**
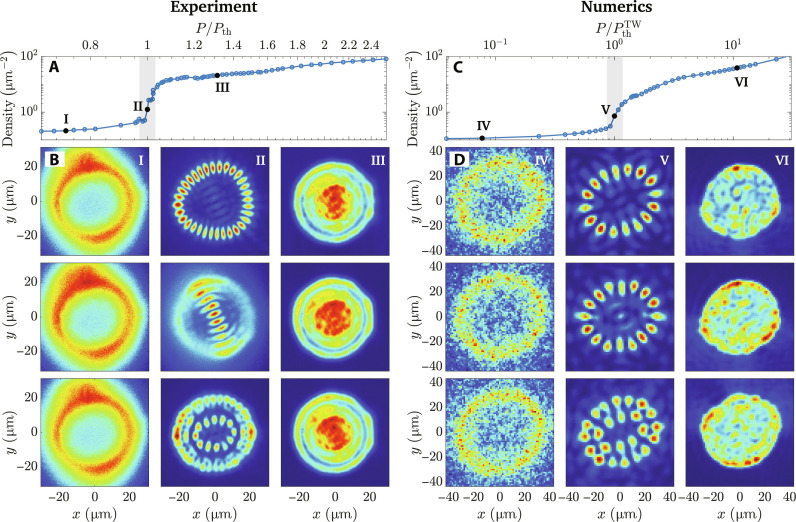
Critical mode switching of the condensate. Critical mode competition and density patterns in the experiment [left panels (**A** and **B**)] and from theoretical modeling [right panels (**C** and **D**)]. [(B) and (D)] Selected snapshots of the polariton density distributions, where each column corresponds to a fixed pump power below (column I), near (column II), and well above (column III) the condensation threshold. The exact *P*/*P*_th_ for each column is indicated by the black circles in (A) and (C). In (D), the single-realization density profiles are integrated in time after the system has reached the nonequilibrium steady state with the integration period of τ_int_ = 20 ns.

We recorded several snapshots of the PL emission patterns from the trap for different pump powers. Each snapshot was time integrated for 100 ms. Because the pump laser was optically chopped, each snapshot is a sum of 42 images from separate laser pulses, each of which was about 42 μs long. Crucially, the pump conditions are identical for all snapshots at each pump power because we used a stabilized M Squared Laser, with very low noise. Figure 2A shows the density across the noncondensed-to-condensed transition as the pump power was increased; in Fig. 2B, we show different snapshots of the PL in real space below at and above the threshold of condensation. Below the threshold, polaritons remain in the vicinity of the pump spot and the PL looks spatially similar from one snapshot to the next as shown in Fig. 2B, column I. For pump power above the condensation threshold, the polariton population builds up inside the trap. When the density becomes larger than the critical density, stimulated scattering becomes significantly enhanced, giving rise to a condensate mode. These modes have been seen in previous experiments with photonic detuning (*33*–*38*) and resemble pure lasing modes in VCSELs with imposed circular symmetry (*24*–*27*).

As expected, near the onset of condensation, we observe strong density fluctuations. Some of these fluctuations can be attributed to hopping of the condensate between different discrete eigenstates. Although the pump conditions are identical from one snapshot to the next, the polariton gas near the threshold of condensation does not always condense to the same mode but rather switches between several distinguishable modes as illustrated in Fig. 2B, column II. Although each snapshot is the result of summing 42 images from separate laser pulses, statistical clumping of certain modes—in small samples of 42—is able to reproduce the same mode switching effect (see Supplementary Materials for more details). Notably, unlike optical multistability or bistability mechanisms, which can take place under resonant excitations at higher pump powers (*39*, *40*), the critical fluctuations we see are only present at the condensation threshold. Unexpectedly, we find that the time scale of the fluctuations is very long compared to the intrinsic scattering time scale of the polaritons; features are seen to differ in images recorded with time integrations of 100 ms. This indicates that the system has metastable telegraph-type switching between modes, as has been seen for lasers (*41*–*43*). Here, this corresponds to the mode competition in the condensate formation process. In equilibrium BEC, it is the ground state of the system, which becomes macroscopically occupied. Because our system is driven-dissipative and out of equilibrium, the process of condensate formation is more complex. Which mode wins out to condense depends on the subtle interplay of gain and interaction effects. Eventually, as the pump power is increased away from the condensation threshold, the density fluctuations become less relevant and the polariton fluid occupies one mode for all different snapshots (see Fig. 2B, column III).

### Theoretical modeling

Near the critical threshold of condensation, mean-field (MF) theories are known to become inaccurate because the fluctuations of the order parameter about its mean value become large, and the state of the many-body system is no longer accurately described by a single-particle wave function. To model the experimental results, we describe the strongly fluctuating polariton field within the truncated Wigner (TW) approximation (*44*, *45*) in terms of a stochastic equation coupled to a rate equation for the excitonic reservoir. The full model and further details regarding numerical methods can be found in Materials and Methods.

We solve the stochastic equations with a Wiener noise at each time step for many realizations of the initial noise. Both initial noise and the noise during the time evolution simulate quantum fluctuations. We emphasize that the amplitude of the Wiener noise in the system is not tuned externally, but rather it is introduced self-consistently in the model. To simulate the experimental measurements, the polariton dynamics is left to evolve for a long time (up to 0.5 μs) after the nonequilibrium steady state (NESS) is reached. Then, each realization is independently integrated over different time windows of the NESS. In Fig. 2, we report typical results from our model. While the behavior of the polariton density is depicted in Fig. 2C, typical steady-state realizations are plotted in Fig. 2D, for three cases: below, near, and above the critical point (Fig. 2D, columns IV, V, and VI, respectively). Similar to the experimental cases explored in Fig. 2, A and B, below and far above the threshold of condensation, the quantum noise plays no role, producing an ensemble of similar images for each realization (Fig. 2C, columns IV and VI). Close to the threshold, however, the quantum noise becomes important, giving rise to realizations with different modes (Fig. 2D, column V).

### Measuring critical mode competition

**Fig. 3.**
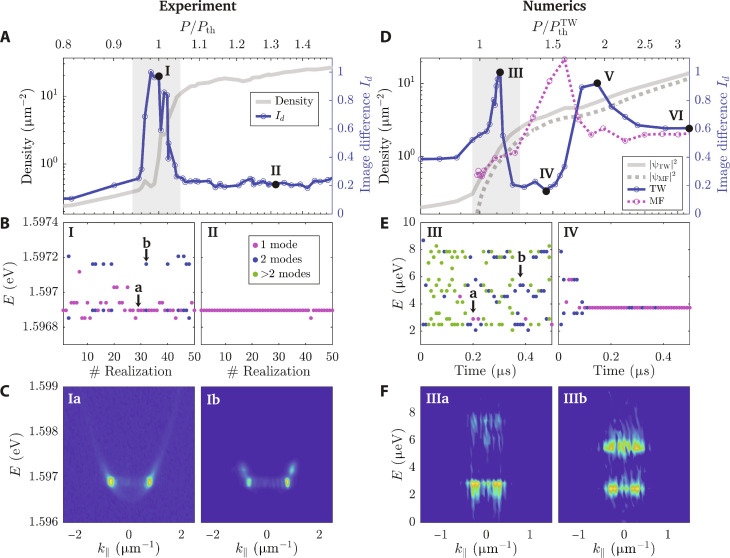
Image difference, energy hopping, and spectra from the experiment and theoretical model. (**A**) Normalized average image difference *I_d_* given by Eq. 1 (blue circle and blue solid curve) and the polariton density (gray solid line) as a function of *P*/*P*_th_. (**B**) Extracted energy peaks for each snapshot, (I) near and (II) far from the critical point. In some cases, the condensate is single mode (purple dots), while in others, it is multi-mode (blue dots). (**C**) Representative *I*(*k*, *E*) snapshots from which the energy peaks were extracted. We show two examples for a single-mode (Ia) and a multi-mode state (Ib) close to criticality. (**D**) Same as in (A), but from theoretical MF and TW analysis. Polariton densities are plotted with dashed (solid) gray line for MF (TW). The average image difference *I_d_* is shown as magenta (blue) circles and magenta (blue) solid line for MF (TW). In (A) and (D), the critical region is marked as a gray shaded area, consistent with previous work (*55*). The threshold power was identified where the plot changes slope in a log-log scale. (**E**) Extracted energy peaks along the time evolution of a single realization of the models (two and three), within (III) and above (IV) the critical region. (**F**) Same as (C), but for the theoretical analysis showing two time-integrated snapshots with different number of modes.

To gain a quantitative description of the critical fluctuation features exhibited by the polariton system, we calculated the normalized average image difference between different consecutive images from the experiments for a given set of parameters such as pump power. This can be written as

$$I_{d}=\frac{\left\langle \text{RMS}\left(I_{n+1}-I_{n}\right)\right\rangle }{\left\langle I_{n}\right\rangle }$$ (1)

where *I_n_* is the *n*th intensity image in real space and RMS stands for root mean squared. This average image difference is then normalized for each pump power by the average intensity for that set. We plot this quantity for both the experiment and the numerical simulations in Fig. 3 (A and D, respectively). While relatively small below and above the critical point, the average image difference *I_d_* is found to show a maximum exactly at the threshold region, indicating that fluctuations are large near the condensation threshold. To verify that this behavior is due to the onset of competition between different modes, we make use of angle-resolved imaging to access the information in momentum space (*46*). Such a procedure allows us to extract the number of modes, together with their energy, over the whole set of images. In Fig. 3B, we plot energy hopping for powers at and above the critical threshold, revealing the single mode to many-mode transition. The quantity *I*(*k*_∥_, *E*) is shown in Fig. 3C for two typical configurations: single-mode (panel Ia) and multi-mode condensate (panel Ib).

Our numerical simulations are in good agreement with the experiment; the numerical average image difference has a maximum near the threshold (point III in Fig. 3D), while it markedly decreases at lower and higher pump powers (point IV). We can extract the different mode energies by Fourier transforming the computed wavefunction ψ = ψ(*r*, *t*) to ψ = ψ(*k*, ω). Experimentally, we did not measure the energy fluctuations in real time. Instead, we extracted the mode energy for each snapshot, allowing us to plot the energy as a function of snapshot number. We can, however, access this information from our numerical simulations; transforming over different temporal windows, we extract the energy peaks for each spectra and plot the energy as a function of time (Fig. 3E).

### Mode hopping during relaxation

**Fig. 4.**
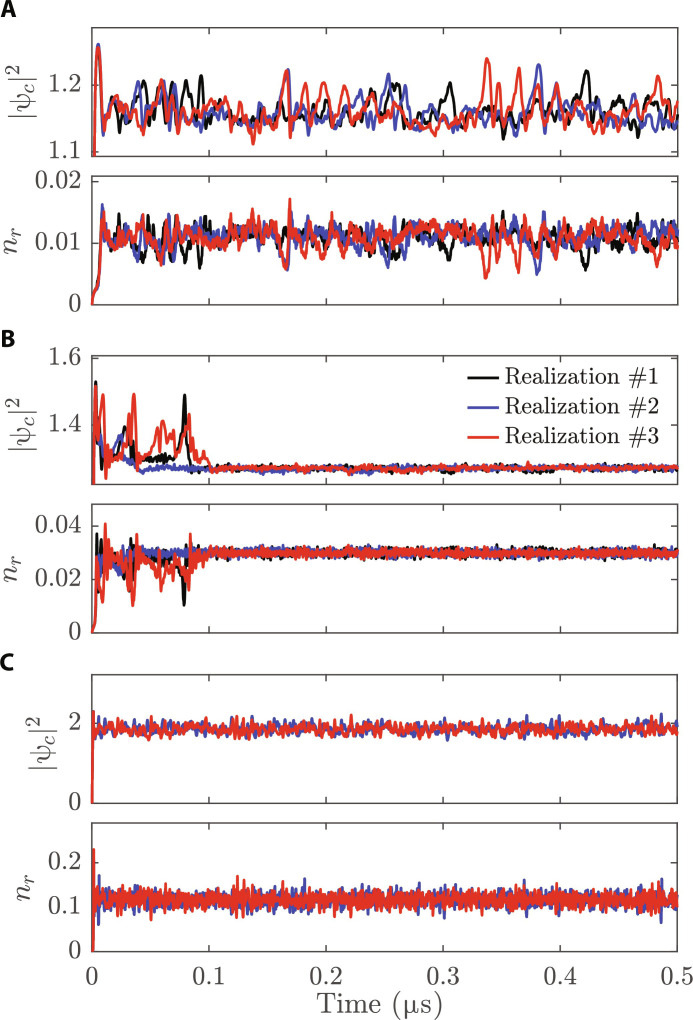
Density evolution. The simulated total condensate ∣ψ*_c_*(*t*)∣^2^ and reservoir *n_r_*(*t*)^2^ densities for (**A**) $P/P_{\text{th}}^{\text{TW}}=1.12$ , (**B**) $P/P_{\text{th}}^{\text{TW}}=1.44$ , and (**C**) $P/P_{\text{th}}^{\text{TW}}=3.2$ , corresponding to III, IV, and VI of Fig. 3D, respectively. The different curves (black, blue, and red) represent three different realizations. Density is in μm^−2^ units.

Let us now focus on the mode hopping behavior of the different cases shown in Fig. 3E. At pump powers well above the threshold (Fig. 3E, IV), the system undergoes energy fluctuations and hopping during only the early dynamics of the system and then settles down to a single mode. Such a behavior is linked to the relaxation dynamics expected to take place in coupled condensate-reservoir systems. In previous work, the relaxation dynamics and the dynamical instabilities have been found to be a direct effect of the polariton-reservoir repulsive interaction (*47**–**51*), the latter being closely related to the polariton hole-burning effect (*45*). In Fig. 4B, corresponding to the case above the threshold (point IV in Fig. 3D), the strong density fluctuations take place up to time 20 < τ_RD_ < 100 ns before eventually relaxing down. Note that at higher pump powers (Fig. 4C), corresponding to point VI in Fig. 3D, the NESS is reached after a very short relaxation dynamics with characteristic time of the order of a few nanoseconds.

### Critical mode competition

At pump powers close to the condensation threshold, the situation changes substantially. As depicted in Fig. 3E (panel III), the system spends time in three or more distinguishable states along its entire dynamics. Figure 4A shows that the mode competition leads to strong density fluctuations. In Fig. 3F, we plot the *I*(*k*, *E*) spectra of two typical modes (further details on the spectral analysis of the mode competition are reported in the Supplementary Materials). This numerical observation helps us to give a quantitative picture of the system’s behavior at the critical point: Different stochastic realizations (initialized with different numerical seeds) lead to very different and independent dynamics. Each stochastic path exhibits strong fluctuations in density and hopping between different modes and energies. Note here that these fluctuations persist for time scales up to ∼20 ns, which are orders of magnitude larger than the polariton lifetime, leading to persistent “intermittent” oscillations, which resemble telegraphic behavior observed in photonic systems (*24*–*27*). We note that, as opposed to the configurations at larger pumps discussed in the previous section, the fluctuations persist over the whole NESS dynamics.

While one would expect that by time-averaging over long periods, these fluctuations would eventually be washed out, we find that imaging of mode competition is accessible even when integrating up to times comparable to the NESS duration (see fig. S6 and discussion in the Supplementary Materials). The mode-competition effect can therefore be measured by making use of such “statistical clumping” of certain modes in small ensembles of measures; we discuss this in more detail in the Supplemental Materials. We also investigated the effects of disorder on the mode hopping. Introducing a static disorder profile, with amplitude and spatial correlations matching the experiment, we observe an enhancement of the image difference *I_d_*. A more detailed discussion is reported in the Supplementary Materials.

Last, we note that in the experiment, the measured time scales of the order of a few microseconds are longer than the ones observed in the theoretical analysis. We find that such a discrepancy can be explained by introducing low frequency pump modulations, mimicking external perturbations that are intrinsic to the experimental setup, but not accounted for in the earlier theoretical modeling. We analyzed how these time scales depend on external perturbations; our results are discussed in the Supplementary Materials. We introduced small periodic modulations to the pump of the order of the time scale of the numerical mode hopping (∼20 ns). We observed that the switching time scales can be extended up to the total duration of the numerical dynamics, i.e., on the order of microseconds, therefore matching the switching time scales of the experimental measurement.

### Quantum fluctuations versus classical noise

We now discuss the role of quantum noise at the onset of critical mode competition. We can see the importance of quantum fluctuations by comparing the numerical simulations in the beyond MF TW picture with the same model treated at the MF level. In the latter case, to account for classical variations in the driving strength, we include real-valued spatiotemporal fluctuations to the driving profile. Details of both models are reported in the Material and Methods.

We can calculate the quantity *I_d_* from the outcomes of the two models, which are plotted in Fig. 3D as a blue solid curve (TW) and a pink dashed curve (MF). Let us now restrict ourselves to the critical region, corresponding to the gray shadowed area in Fig. 3D. While the TW (blue) curve exhibits a large peak in the vicinity of the critical point, which results from the mode competition physics discussed in the previous sections, the MF (pink) curve shows an absence of such a peak. The peak near the threshold of condensation can be reproduced only by the self-consistent quantum fluctuations accounted within the TWA. Inspection of the density profiles in the two cases confirms the absence of mode hopping in the latter case. We demonstrate here that fluctuations in both density and phase are essential to explain the critical mode hopping; “classical” amplitude fluctuations in the pump strength alone are not sufficient. While we cannot account for all potential sources of classical fluctuations (e.g., phonons and high-energy excitons), we believe that they are orders of magnitude weaker than the quantum noise coming from photon decay and pumping (see the Supplementary Materials for further discussion).

### Dynamical instabilities at larger pumps

While we have focused so far on the critical region, it is interesting to also comment on the physics taking place at higher pump powers (i.e., higher polariton densities). In Fig. 3D, we note the presence of a second peak at $P/P_{\text{th}}^{\text{TW}}∼1.92$ , i.e., point V. Our investigation of the polariton and reservoir density profiles suggests that this peak originates from the mechanism of dynamical instability (*52*, *53*). As discussed in more detail in the Supplementary Materials, these can be reproduced by moving to a MF formulation of Eqs. 2 to 3 while adding classical-noise variations to the pump profile (pink dashed curve of Fig. 3D). These instabilities are found to be responsible for the large peak in the MF curve too. At very large pump powers ( $P/P_{\text{th}}^{\text{TW}}∼3$ ), the quantity *I_d_* eventually decreases. The density profiles shown in Fig. 2D reveal that at point VI in Fig. 3D, the image difference is due to strong density fluctuations extended over the whole confined region; a different behavior when compared to the critical mode competition observed at point III. We expect that these density fluctuations would eventually smooth out and resemble more the experimental profiles when considering multiple time windows of the order of tens of microseconds as in the experiment.

## DISCUSSION

In this work, we investigated experimentally and numerically the density fluctuations in a circularly confined nonequilibrium BEC of polaritons. As in some experiments with mode hopping in lasers, at the vicinity of the critical region, we observe “telegraphic” switching with long periods of metastability. While the phenomenon of mode hopping is a standard feature in photonic systems such as lasers and VCSELs (*24*–*27*), in this work, for the first time, we are able to demonstrate that in the photonic limit of an interacting polariton fluid, the mode competition arises from the interplay between interactions and quantum noise. Our results suggest that, analogously to other activation problems, quantum fluctuations are responsible for switching between different states.

In addition, we distinguish between the mode competition at the critical point and the relaxation mechanisms expected to take place at larger drivings. We explore in detail the effects of external perturbations and the role of dynamical instabilities at larger pump powers. Our numerical analysis demonstrates that beyond MF quantum effects are crucial for the onset of mode competition and that it cannot be replicated by a model with just classical noise (i.e., fluctuations in the pump power). These findings are crucial for understanding of critical density fluctuations in confined quantum systems. Our work paves the way for further investigation of the mechanism of condensate formation in both equilibrium and nonequilibrium settings.

## MATERIALS AND METHODS

### Sample design

The microcavity used in this work consists of a total of 12 GaAs QWs (7 nm thick) with AlAs barriers embedded between two DBR mirrors, which together form an optical cavity (Fig. 1A). The DBRs are made of alternating layers of AlAs and Al_0.2_Ga_0.8_As. The top DBR is composed of 32 pairs, and the bottom DBR is composed of 40 pairs. The QWs are in groups of four, with each group placed at one of the three antinodes of the 3λ/2 cavity. The large number of DBR periods gives the cavity a high *Q*-factor, resulting in a cavity lifetime of ∼135 ps and a polariton lifetime of ∼270 ps at resonance (*28*–*30*). The long cavity life time allows polaritons to propagate over macroscopic distances of up to millimeters. The wavelength of the pump laser was tuned to the second reflective minimum (725 nm), about 113 meV above the lower polariton resonance.

### Numerical model and parameters

Within the TW approximation (*44*, *45*), the strongly fluctuating polariton field can be described in terms of a stochastic equation of motion coupled to the rate equation for the excitonic reservoir, which reads

$$\begin{array}{c} i\hbar d\psi \left(r\right)=\mathrm{dt}\{\left(i\beta -1\right)\frac{\hbar^{2}\nabla^{2}}{2m}+g_{c}{|\psi \left(r\right)|}_{w}^{2}+g_{R}n_{R}\left(r\right)+\\ +\frac{\mathrm{ih}}{2}\left[{\mathrm{Rn}}_{R}\left(r\right)-\gamma_{c}\right]\}\psi \left(r\right)+i\hbar {\mathrm{dW}}_{c} \end{array}$$ (2)

$$\frac{d}{\mathrm{dt}}n_{R}\left(r\right)=P_{r}\left(r\right)-\left[\gamma_{R}+R|\psi \left(r\right){|}_{w}^{2}\right]n_{R}\left(r\right)$$ (3)

where ψ(**r**, *t*) is the polariton field, *n_R_*(**r**, *t*) the excitonic reservoir density, and *m* the polariton mass. γ_c_ and γ_R_ define the decay rates of condensed polaritons and the excitonic reservoir respectively. Following (*45*), the former term can be estimated as γ_c_ = (1 − ∣*X*∣^2^)γ_ph_, where γ_ph_ = 1/τ_ph_ corresponds to the inverse of the photon lifetime and *X* is the Hopfield coefficient (*54*). The excitonic fraction ∣*X*∣^2^ is given by ${\left|X\right|}^{2}=1/2\left(1+\delta /\sqrt{4\hbar^{2}\Omega^{2}+\delta^{2}}\right)$ , where Ω is the Rabi splitting and the detuning δ corresponds to the difference between the photonic and excitonic energies. In Eq. 2, the Wigner noise accounts for quantum fluctuations and is space- and time-correlated as $<dW_{c}\left(r,t\right)dW_{c}(r\prime ,t)>=0$ , $<dW_{c}\left(r,t\right)dW_{c}^{*}\left(r\prime ,t\right)>=[\gamma_{c}+Rn_{R}\left(r\right)]/2\;\delta_{r,r\prime }\mathrm{dt}$ . Moreover, the renormalized density ${|\psi |}_{w}^{2}\equiv {|\psi |}^{2}-1/2a^{2}$ includes the subtraction of the Wigner commutator contribution, where *a* is the numerical two-dimensional (2D) grid lattice spacing. The relaxation parameter β sets the amount of energy relaxation and increases as the detuning δ = *E*_c_ − *E*_ex_ increases. The constants *g*_c_ and *g*_R_ are the strengths of polariton-polariton and polariton-reservoir interactions, respectively; they can be estimated as *g*_c_ = *g*_ex_ ∣*X*∣^4^, *g*_R_ = *g*_ex_ ∣*X*∣^2^, with *g*_ex_ the exciton-exciton interaction. The parameter *R* = *R*_0_*g*_c_/*g*_R_ quantifies the stimulated scattering rate of the reservoir excitons into the polariton condensate (*45*). To match the experimental geometry, the pump power profile *P_r_*(***r***) = *P*(γ*_c_*/*R*) exp [−( ɛ/2σ^2^)^2^] is modeled as a tilted elliptical geometry, with a rotated axis of angle θ. ɛ is the tilted ellipse equation ɛ = *Ax*^2^ + *Cy*^2^ + *Bxy* − 1, where *A* = (cos^2^θ)/*a*^2^ + (sin^2^θ)/*b*^2^, *B* = 2sinθcosθ and *C* = (sin^2^θ)/*a*^2^ + (cos^2^θ)/*b*^2^, with *a* and *b* the major and minor axis respectively. The Eqs. 2 and 3 are solved numerically adopting an explicit Runge-Kutta method of orders forth and fifth on a 2D numerical grid with *N* = 128^2^ points with grid-spacing *a* = 1.17 μm. We solve the dynamical equations with the following experimental parameters: *m* = 4.2 × 10^−5^*m_e_* with *m_e_* the electron mass, τ_ph_ = 135 ps, γ_R_ = 10^−3^ps^−1^, *g*_c_ = 2 μeVμm^2^, *R*_0_ = 2 × 10^−3^μm^2^ps^−1^, σ = 1.53 μm, *b* = 30 μm, *a* = 1.1*b*, and θ = 0.5.

### MF with classical fluctuations in the pump

In the main text, we discuss the role of quantum fluctuations in the mode competition by comparing the results obtained from the TW equations, i.e., Eqs. 2 and 3, with their MF approximation and only a classical noise (introduced as fluctuations in the pump power). The polariton equations of motion in the MF approximation read

$$\begin{array}{c} i\hbar d\psi =\mathrm{dt}[\left(i\beta -1\right)\frac{h^{2}\nabla^{2}}{2m}+g_{c}|\psi {|}^{2}+g_{R}n_{R}+\\ +\frac{i\hbar }{2}\left(Rn_{R}-\gamma_{c}\right)]\psi \end{array}$$ (4)

$$\frac{d}{\mathrm{dt}}n_{R}={\tilde{P}}_{r}\left(r\right)-\left(\gamma_{R}+R|\psi {|}^{2}\right)n_{R}$$ (5)

To explore the effects of classical fluctuations in the driving, we introduce spatial-temporal variation in the pump power

$${\tilde{P}}_{r}\left(r\right)=\left[1+\chi \mathrm{dW}\left(r,t\right)\right]P_{r}\left(r\right)$$ (6)

where *dW*(**r**,   *t*) is a zero-mean, real-valued Gaussian noise both in space and time. This formulation excludes the nonclassical correlations present in the TW approximation. Here, the term χ quantifies the amount of classical fluctuations on top of the driving pump *P_r_*(**r**).

In typical experimental setups, the strength of the classical noise is usually ≈1% of the pump intensity. In our numerical analysis, we push the strength of the noise up to 10% of the total intensity of the pump, corresponding to χ = 0.1. Even at these strong classical noise amplitudes, we do not see any peak around the threshold $P_{\text{th}}^{\text{TW}}\approx 1$ on the *I_d_* purple curve plotted in Fig. 3D, confirming that the *I_d_* peak measured at criticality is due to the presence of the Wigner quantum noise included in the TW approximation, as discussed in the main text. We refer the reader to the Supplementary Materials for further numerical details.

## Supplementary Materials

### This PDF file includes:

Supplementary Text Figs. S1 to S8 References
