# Supplementary material for: Critical fluctuations in a confined driven-dissipative quantum condensate
Source: Sci Adv. 2024 Mar 22;10(12):eadi6762. doi: 10.1126/sciadv.adi6762 (PMC10959404; doi:10.1126/sciadv.adi6762)
Supplement: Supplementary file 1 — Supplementary Text Figs. S1 to S8 References [file sciadv.adi6762_sm.pdf]

Science Advances
AAAS

Supplementary Materials for

Critical fluctuations in a confined driven-dissipative quantum condensate

Hassan Alnatah *et al.*

Corresponding author: Hassan Alnatah, haa108@pitt.edu

Sci. Adv. **10**, eadi6762 (2024)
DOI: 10.1126/sciadv.adi6762

This PDF file includes:

Supplementary Text
Figs. S1 to S8
References

I. SWITCHING DYNAMICS

As explained in the main text, experimentally, we did not measure the energy fluctuations in real time. Instead, we took many snapshots of the polariton gas using angle-resolved imaging for each pump power and obtained the mode energy for each snapshot image $I(k,E)$. This was extracted by first summing the image $I(k,E)$ horizontally (i.e. integrating over k) to obtain the intensity $I(E)$, and then numerically extract the energy for each $I(E)$ peak as shown in Fig. S1 **(B)**. The columns in Fig. S1 from left to right relate to below, near and above the threshold of condensation (which correspond to points I, II, and III in Fig. 2 of the main text). We then plot the extracted energy as a function of the snapshot number (Fig. S1 **(C)**). To help visualize the frequency of each mode, we use the points in Fig. S1 **(C)** to create a histogram as shown in Fig. S1 **(D)**. We find that the polariton gas is multimode near the condensation threshold and single mode away from the condensation threshold.

We follow a similar procedure for the numerical data, which allows us to extract the energy fluctuations (Fig. S2). Figure. S2, columns II, III, V, and VI correspond to points III, IV, V and VI of Fig. 3 of the main text, respectively. These points are discussed in the main text and here we include the full data for completeness. We first integrate the real space solution $\psi(r,t)$ over a 20 ns temporal window. The real space wavefunction $\psi(\boldsymbol{r})$ is then Fourier transformed to $\psi(\boldsymbol{k})$. $|\psi(\boldsymbol{r})|^2$ and its Fourier transform $|\psi(\boldsymbol{k})|^2$ are shown in Fig. S2**(A)** and Fig. S2**(B)** respectively for the last 20 ns temporal window of the dynamics. To find the energy resolved spectra, we Fourier transform the wavefunction $\psi = \psi(\mathbf{r},t)$ to $\psi = \psi(\mathbf{k},\omega)$ over the same 20 ns temporal window. We then follow the same method mentioned earlier to extract the energy fluctuations by extracting the energy peaks from the numerical $I(k,E)$ images (see Fig. S2**(D-F)**). We find that below the condensation threshold, there is a continuum of energy states (see Fig. S1 **(E)**, column I). Near the critical regime, the condensate switches between three main states with almost equal distribution (see Fig. S2 **(F)**, column II). Importantly, these critical fluctuations persist over the total simulated time as shown in Fig. S1 **(E)**, column II. Well above the threshold, the condensate undergoes switching during early dynamics and then settles down to a single mode (Fig. S1**(E)**, column III). Since the switching happens only during early dynamics, the histogram shows a single dominant mode as illustrated in Fig. S1**(F)**, column III. Figure. S1**(E)**, columns V and IV show the effect of the dynamical instability (a term which refers to a spatially fragmented condensate, caused by effective attractive interactions, mediated by the excitonic reservoir [50,56]) taking place at higher pump powers. In these cases, the condensate density starts to build up in the centre of the ring, over the whole confined region. What we then see are the spatial fluctuations in the condensate density (spatially non-uniform/fragmented condensate) in a single energy mode. This effect is different from the one observed at criticality; a detailed explanation of this phenomenon is given in the next sections.

II. STATISTICAL CLUMPING

The experimental emission patterns in Fig. 2 of the main text are a sum of 42 images from separate pulses, while in the simulations each realization corresponds to a single pulse. In this section, we show that the same mode hopping effect can be reproduced by statistical clumping of certain modes in small samples of 42. To model the statistical clumping, we first start with three modes. We then randomly choose one of these modes N_{pulse} times and compute the sum of these randomly chosen N_{pulse} modes. This sum of the N_{pulse} images is therefore equivalent to a snapshot in the experiment (in the experiment, $N_{\text{pulse}} = 42$). Experimentally, we recorded 300 snapshots for each pump power. Therefore, we repeat the above process in the simulations to generate 300 snapshots. We then use Eq. (1) from the main text to compute the average image difference of these snapshots. To illustrate the significance of statistical clumping, we vary N_{pulse} (i.e the number of images that are being summed over) and we plot the image difference as a function of N (see Fig. S3). As seen in Fig. S3, statistical clumping of three modes in a small sample of 42 laser pulses (the red circle shown in the plot) shows a noticeable image difference. As expected, when the number N_{pulse} becomes very large, the image difference tends to zero. However, the experimental regime is in small samples of $N_{\text{pulse}} = 42$, where mode-hopping can still be observed. We find that the image difference I_d gets reduced by roughly 0.2 when $N_{\text{pulse}} = 42$ compared to the case of $N_{\text{pulse}} = 1$. Therefore, the effect of summing over images from separate pulses reduces I_d but the overall mode hopping effect can still be observed in a small sample of $N_{\text{pulse}} = 42$.

Although the results we have presented in Fig. 2 of the main paper show the condensate in only a single mode, very often we also observe the polariton gas in a linear combination of these modes. However, since we take many snapshots of the condensate, occasionally we also observe it in a single dominant mode even after averaging over 42 chopped pulses. Figure S4 shows an example of polariton condensate in a single mode (left and middle panels) and a linear combination of these modes (right panel) for the same pump power $P/P_{th} = 1$.

III. ENERGY SPACING

In the experiment, we observed that energy spacing between the switching modes of the system is about 200 μeV, while in the simulations, the observed energy spacing is 2 μeV. In this section, we show that in the simulations the switching happens between states with energy close to the ground state of the system, while ex-

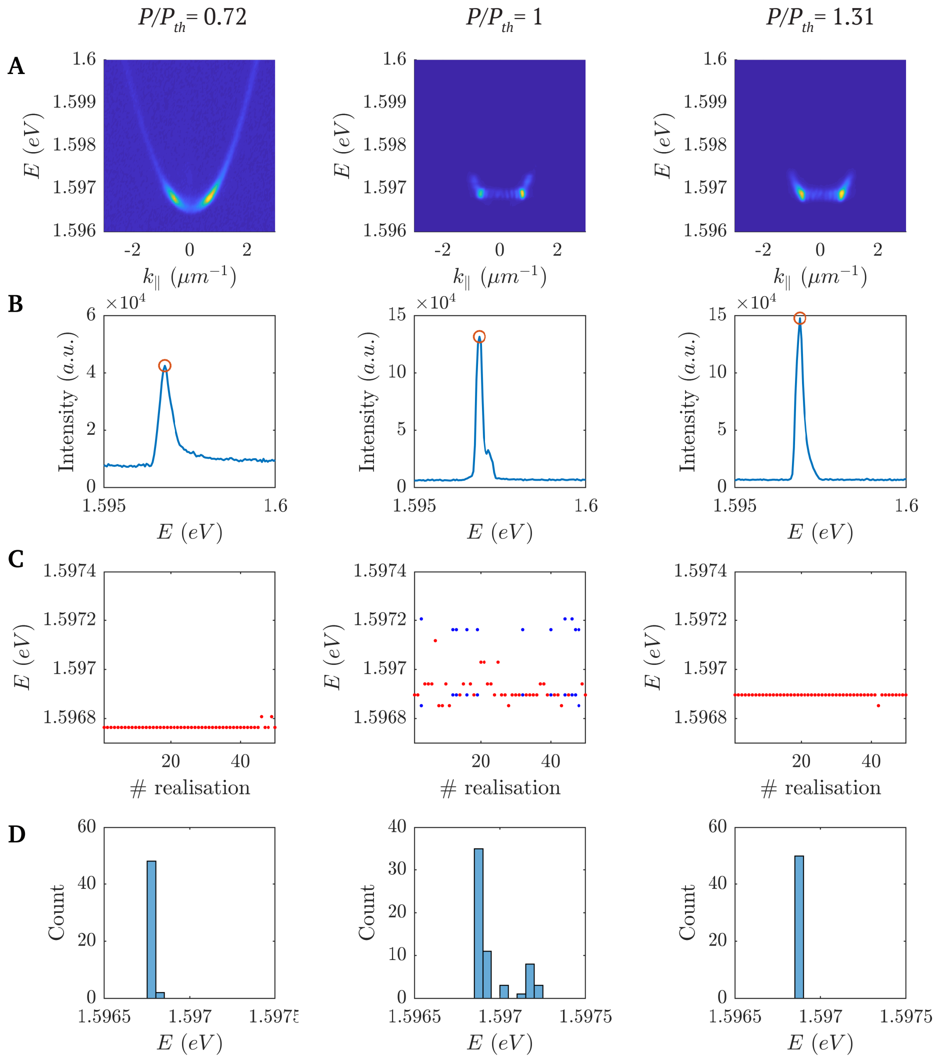

Figure S1: **Experimental energy analysis.** **(A)** Representative $I(k, E)$ snapshots from which the energy maxima were extracted. **(B)** $I(E)$ obtained by horizontally summing $I(k, E)$. The red circles illustrate the energy peaks extraction. **(C)** Switching dynamics for each snapshot. **(D)** A histogram of the occurrence of modes with given energy. The first column corresponds to below, the second to near, and the last column to above the condensation threshold.

perimentally the switching occurs between higher energy modes.

To find which of the single particle states the condensates chooses, we solve the Schrodinger equation without nonlinearities in 2D with an annular potential.

$$-\frac{\hbar^2}{2m_{\text{pol}}}\nabla^2\psi(\boldsymbol{r}) - V(\boldsymbol{r})\psi(\boldsymbol{r}) = E\psi(\boldsymbol{r}). \quad \text{(S1)}$$

We then compare which of the solutions $|\psi_{n,m}(\boldsymbol{r})|^2$ look spatially similar (i.e. the same number of petals or ripples) to the experimental and theoretical real-space images. The eigenstates are calculated using a potential of the form $V(r) = V_0 \exp\left[-(r - r_0)^2/2\sigma\right]$, where r_0 is the radius and σ is the width of the potential ring. We solved Eq. (S1) numerically to find $|\psi_{n,m}(\boldsymbol{r})|^2$ and $E_{n,m}$. The quantum number n determines the number of nodes in the radial direction, while m determines the nodes around the ring. The number of radial nodes is given by $n - 1$ and the number of nodes around the ring is given by $2m$.

We find that in the experiment, the condensate switches between three main modes $\psi_{(n=1,m=16)}$, $\psi_{(n=5,m=1)}$ and $\psi_{(n=3,m=16)}$, which are shown in Fig. 2, column II of the main text. In the simulations, the switching happens between modes $\psi_{(n=1,m=8)}$, $\psi_{(n=2,m=8)}$ and $\psi_{(n=1,m=1)}$. The energy spacing is roughly proportional to $z(m_{\text{final}}, n_{\text{final}})^2 - z(m_{\text{initial}}, n_{\text{initial}})^2$, suggesting that switching between higher energy modes will give larger energy spacing, where $z(m, n)$ is the n-th zero of the regular Bessel function $J_m(z)$. Of course, the real-

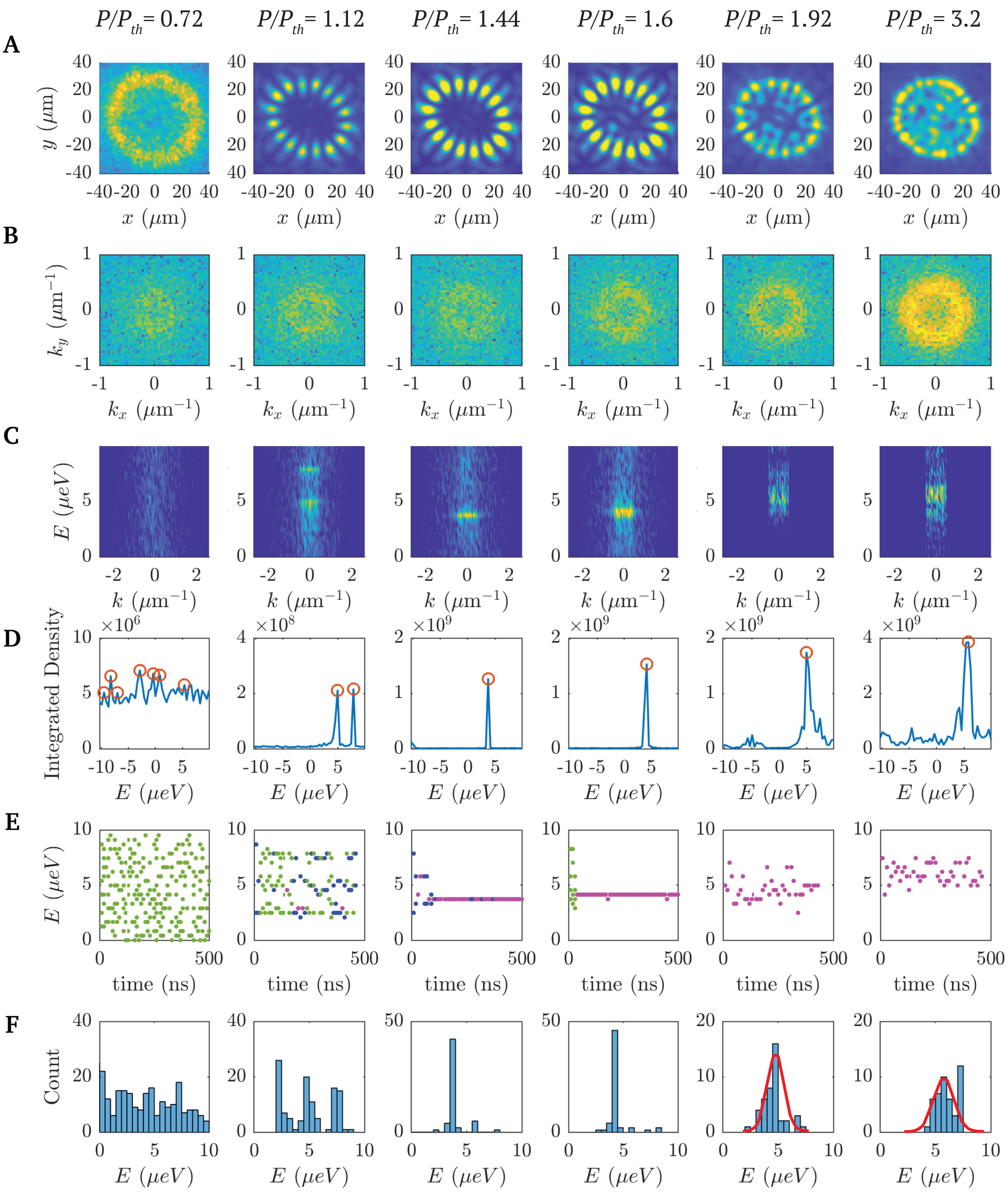

Figure S2: **Theoretical energy analysis.** **(A)** Real space $|\psi(\boldsymbol{r})|^2$ and **(B)** momentum space $|\psi(\boldsymbol{k})|^2$ densities. **(C)** $|\psi(\mathbf{k}, E)|^2$ from Fourier transforming $\psi = \psi(\mathbf{r}, t)$ to $\psi = \psi(\mathbf{k}, \omega)$. In **(A-C)** we have integrated over the last 20ns of the dynamics. **(D)** $I(E)$ from integrating $I(k, E)$ over k. The red circles illustrate the energy peaks extraction. **(E)** The energy peaks as a function of time. We periodically sample different temporal windows of 20 ns. Colors of the dots correspond to: single mode (purple); two modes (blue); three and more modes (green). **(F)** A histogram of the number of realisations within given energy interval. The red Gaussian curves in the last two histograms are a guide to the eye.

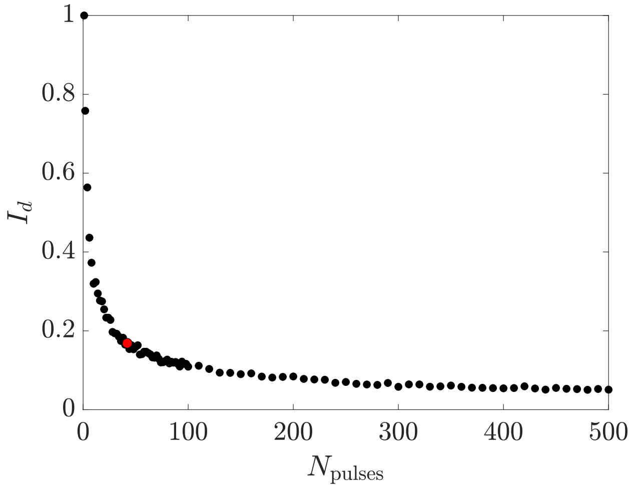

Figure S3: **Statistical clumping of modes.** Normalized image difference by randomly choosing 3 modes N_{pulses} times, and summing over them. This process is repeated 300 times, each corresponding to a single snapshot. We use Eq. (1) in the main text to compute I_d . The red circle at N_{pulses} = 42 shows the regime we work with experimentally.

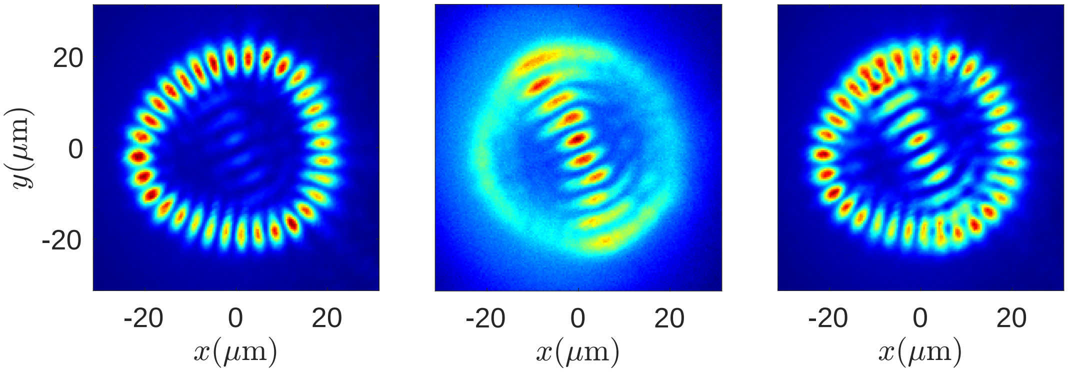

Figure S4: **Linear combination of modes.** Multiple snapshots of the experimental polariton density profile in real space taken at the threshold power. In the left and middle panels, the condensate is single mode while for the right panel, the condensate is in a linear combination of these two modes.

space images of the condensate both in the experiment and the simulations include nonlinearities. However, the solution to the linear Schrodinger equation still allows us to find the quantum number of the single particle states, which participate in condensation. The states obtained from solving the Schrodinger equation are close to the real space distributions obtained from the experiment and the simulations with only small deviations due to relatively weak polariton interactions.

In our numerical simulations, we find that the modes, which participate in the mode switching process, depend on various parameters of the model such as the relaxation and interaction strengths. The values of these parameters are not exactly known for semiconductor microcavities [57]. Exploring in principle a large range of possible parameter space is both time-consuming and computationally expensive. We, therefore, focus on reproducing the experiment qualitatively i.e. showing that the mode-hopping is pronounced near the threshold of condensation and is greatly suppressed away from it.

IV. DENSITY CALIBRATION

In Fig. 2(**A**) and Fig. 3(**A**) of the main paper, we have used the photon counting method to calibrate the density of the polaritons. Photon counting allows us to relate the number of counts on the CCD camera to the number of photons detected. This was done by matching the laser wavelength to the polariton emission wavelength

(776.5 nm). The laser was sent to a mirror at the sample plane, which reflects the laser through the same optical path that was used in the experiment. This reflected beam was then imaged with the CCD camera. The CCD count is proportional to the number of photons, which can be written as:

$$I_{\rm CCD} = \frac{1}{\eta}\frac{N_{\rm ph}}{\Delta t}, \tag{S2}$$

where Δt is the integration time of the camera, $N_{\rm ph}$ is the number of photons detected and η is the efficiency factor. The number of photons that the camera detects during a time Δt is then:

$$N_{\rm ph} = \frac{P\Delta t}{hc/\lambda}, \tag{S3}$$

where P is the measured power of the laser, h is Planck constant, c is the speed of light and λ is the wavelength of the laser. This then allows us to find a single efficiency factor to convert from CCD counts to number of photons being detected. The efficiency factor is given by:

$$\eta = \frac{P\lambda}{hc}\frac{1}{I_{\rm CCD}}. \tag{S4}$$

This efficiency factor was used to calibrate the total density of the polaritons, which is given by:

$$n_{\rm tot} = \frac{\eta I_{\rm CCD}\tau}{A_{\rm obs}}, \tag{S5}$$

where $I_{\rm CCD}$ is the CCD count, τ is the average radiative lifetime of the polaritons and $A_{\rm obs}$ is the observed area on the sample from which the light was collected. Since the excitation laser beam was chopped in the experiment with a duty cycle $d = 1.7\%$, then the total number of polaritons is

$$n_{\rm tot} = \frac{\eta I_{\rm CCD}\tau}{A_{\rm obs} d}, \tag{S6}$$

where $\tau \approx \tau_{\rm cav}/\left|C_{k_\parallel}\right|^2$. Here $\left|C_{k_\parallel}\right|^2$ is the photon fraction and $\tau_{\rm cav}$ is the cavity lifetime.

V. NUMERICAL ANALYSIS

In the main text, we introduced the numerical model used to simulate the experimental observations, and discussed the mode switching near the phase transition. In this section, we give more details about the numerical analysis; we first discuss the model and the results from the mean-field equations with noise added to the pump profile (Sec. V A). We then explain the effects of the system relaxation dynamics on mode competition (Sec. V B) and finally, we explore the effects of introducing classical external perturbation simulated by a periodic pumping (Sec. V C).

A. Numerical details of the mean-field modeling with a noisy pump profile.

In the main paper, we investigated the role of quantum critical fluctuations on the observation of mode-hopping features in the polariton system by comparing different models. The mean-field model with a fluctuating pump profile is described in the Methods Section of the main text. In this section, we give further numerical details regarding the simulations of this model.

In Fig. S5, we plot the results obtained by solving Eqs. (4)-(5) of the Methods Section in the main text. The nonequilibrium steady state is reached after evolving the system from a random initial condition for 1μs, which we average over $\mathcal{N} = 100$ realizations. The time evolution of the total averaged density $\langle|\psi(t)|^2\rangle_{\mathcal{N}}$ is plotted in the left panel of Fig. S5 for different pump values. Once the system reaches a nonquilibrium steady state, we extract the average steady-state density shown in the top right panel of Fig. S5. We then calculate the image difference I_d by means of Eq. (1) of the main text and plot it in the bottom right panel of Fig. S5, and in Fig. 3**(D)** of the main text.

B. Effects of dynamical relaxations and instabilities on mode-hopping.

We proceed by discussing the effects of the relaxation mechanisms on mode switching. As shown in the main text and in Fig. S2, in certain regimes above the threshold ($P_{\rm th}^{\rm TW} \approx 1.5$) the system is seen to undergo switching between different energy modes during only the early dynamics of the system before settling down to a single mode. Comparison of the time evolution of the polariton and reservoir densities at this particular pump power regime (shown in Fig. (4) of the main text) suggests that this early-time mode competition originates from the relaxation dynamics in the condensate-reservoir coupled system [45,47-50]. As shown in Fig. (4) of the main text, different stochastic realisations (initialized with dif-ferent numerical seeds) lead to different dynamics. Interestingly, both at criticality and at early time of the polariton dynamics above the threshold, the system exhibits "intermittent" oscillations, which resemble a telegraphic behavior. Such intermittency may be an effect of introducing the relaxation term β in the equations of motions (Eqs. (2)-(3) of the main text); preliminary results on the observation of this intermittent behavior has been reported in uniform systems [58]. We leave the numerical investigation of this behavior, for the case of confined systems, for future work.

To investigate further the effect o f t he r elaxation dynamics on the mode switching over the whole phase diagram, we extract the quantity I_d for different integration-time windows, and investigate real-space density distributions of the polariton condensate. In Fig. S6, we plot three different I_d, c alculated w ithin t he T W approxi-

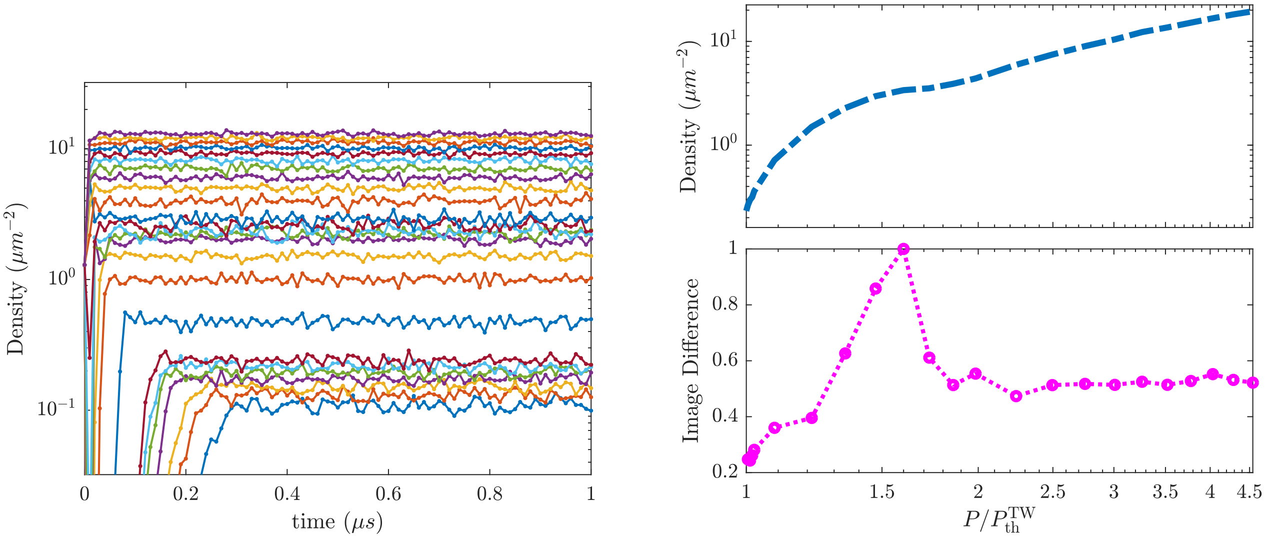

Figure S5: **Mean-field approximation. (Left panel)** Time evolution of the total density $\langle|\psi(t)|^2\rangle_{\mathcal{N}}$ from Eqs. (4)-(5) of the Methods and Material section in the main text averaged over $\mathcal{N} = 100$ realizations, plotted in a linear-log scale. Different colors are for different pump powers as in the right panel. **(Top right panel)** The extracted steady-state densities as a function of pump power normalized to the condensation threshold $P/P_{\mathrm{th}}^{\mathrm{TW}}$ on a log-log scale, the same as the gray point-dashed curve in Fig. 3 of the main text. **(Bottom right panel)** Normalized image difference I_d in a log-linear scale obtained by time-integrating the dynamics shown in the left panel over the interval $0.5\mu\text{s} < T < 1\mu\text{s}$.

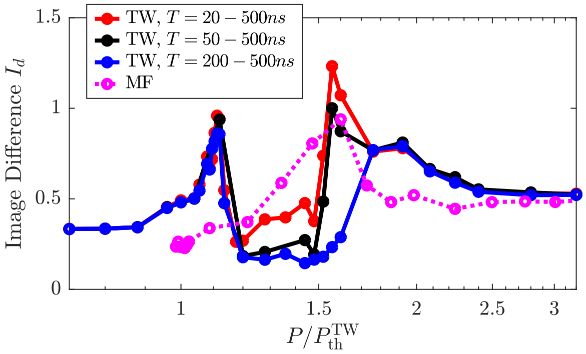

Figure S6: **Image difference.** The image difference I_d, calculated within the Truncated-Wigner model (Eqs.(2)-(3) of the main text) marked as a solid blue line and mean-field approximations (Eqs.(4)-(5) of the main text) marked as a dashed pink line. The same data is shown in Fig. 3**(D)** of the main text. The red and black solid lines correspond to the I_d for the TW model, when integrating over different temporal windows T.

mation, by time-integrating over different temporal windows, from different starting times till the end of the evolution, i.e. $t = 0.5\mu s$. Specifically, we integrate between: $0.02\mu\text{s} < T < 0.5\mu\text{s}$ (red curve); $0.05\mu\text{s} < T < 0.5\mu\text{s}$ (black curve); $0.2\mu\text{s} < T < 0.5\mu\text{s}$ (blue curve). The latter corresponds to the blue curve in Fig. 3**(D)** of the main text. We note that at $P/P_{\mathrm{th}}^{\mathrm{TW}} \approx 1.6$, a large peak appears when considering longer times of integration. Comparison with the density evolution plots in Fig. (4) of the main text suggests that this peak is due to the inclusion of the switching between modes originating from the relaxation mechanisms.

Next, we compare the TW approximation results in Fig. S6 with the image difference I_d, calculated using classical noise added to the pump profile in the MF approximation. (This is shown in Fig. S6 as a pink curve; the same curve is reported in Fig. 3 **(D)** of the main text.) Although the MF results do not exhibit a peak at criticality, as discussed in Sec. V A, it is interesting to note the presence of a maximum at larger pump powers. An investigation of the real-space density images along the time evolution suggests that this is due to the so called modulational instability [50], which is a mean-field effect. Attractive interactions between polaritons, caused by the presence of the reservoir, lead to condensate fragmentation and therefore fluctuating density, which persists over the whole dynamics as shown in the left panel of Fig. S5. This indicates a possible relation between the observed MF peak and the peak at $P/P_{\mathrm{th}}^{\mathrm{TW}} \gtrsim 1.8$ observed in the TW formulation (blue curve).

C. Modeling classical external perturbation with periodic pumping

In the experimental setup, external perturbations e.g. temperature variations or laser modulations, may have a large impact on the critical timescales of the system; such perturbations are not included in our model. In this section, we discuss the role of the dynamics of the external pumping on the switching time-scales of our theoretical modelling. Specifically, our analysis shows that the time-scale of the mode switching can be extended up to the whole dynamics (of the order of microseconds) by introducing small periodic modulations of the amplitude of the pump. We model this phenomenon by means of a periodic pump defined as

$$\mathcal{P}_r(\mathbf{r}) = P(\mathbf{r})\left[1 + A\cdot\sin\left(\frac{t}{\tau_{mod}}\right)\right]. \tag{S7}$$

For our analysis, we choose timescales comparable to the temporal mode-hopping scales of the numerical simulations, namely $\tau_{mod} = 20$ ns. To probe different regimes of modulations, we vary A in the range $A \in [0, 10^{-2}, 10^{-1}]$.

Investigation of the real-space density distributions shows that a choice of $A = 0.01$ extends the switching time scale to over the whole time-dynamics, namely $0.5\mu s$. Moreover, comparison of the quantity I_d between modulated and non-modulated cases ($A = 0$) shows no appreciable differences (< 10%) in the critical region. Indeed, the polariton density follows the modulation of the pump, yet the density profiles do not show large changes when compared to the unperturbed case. To test our results, we run simulations for values up to $A = 0.1$. An investigation of the polariton density distribution shows very large oscillations; moreover, the image difference increases substantially compared to the non-oscillating case.

D. Effect of static disorder on mode switching

We have studied the effect of static disorder on mode switching by introducing a time-independent term $V(r)$ to the Hamiltonian of Eq.(2) of the main text. We assumed that the random potential has a mean amplitude and root mean square fluctuation given by $\langle V(\mathbf{r})\rangle = 0$ and $\sqrt{\langle V^2(\mathbf{r})\rangle} = 10$ μeV respectively. The correlation length of this potential is taken to be 2 μm. We have run 50 different stochastic dynamics with the same random static noise profile but with different random noise in the initial wave function. We then averaged over these 50 different stochastic dynamics and repeated the procedure for 50 different random static noise profiles.

We have found that the static disorder enhances the image difference I_d by a factor of 4. However, we note that the number of modes that undergo mode hopping is still the same when compared to the case with only quantum fluctuations and no static disorder (i.e. the results in the main text shown in Figure 3). Therefore, we conclude that static disorder only introduces density variation of the polariton condensate rather than affecting the number of modes.

E. Convergence Analysis

In this section, we discuss the numerical convergence for the spatial grid spacing a. The TWA method, employed in this work, is computationally very efficient and is able to describe quantum fluctuations. However, TWA has a relatively narrow window of applicability: it can be shown [44] that TWA is valid only for lattice spac-ings greater than the square root of the ratio between interactions and losses: $\gamma_{LP} \gg g_{LP}/a^2$. This condition essentially sets a lower limit for a. This originates from the derivation of TWA equations, where one assumes the 3rd order derivative in Fokker-Plank equation to be small (at least smaller than the 2nd order term which we keep) so that it can be ignored [44].

In our convergence and numerical validity tests, we kept the same set of parameters as in the main text and only varied a and the pump power P. We have run 50 stochastic realizations for different spatial spacing, namely $a = 0.8, 1.02, 1.1, 1.17, 1.55$ μm. We have then investigated the time-averaged density profiles for three different pump power values corresponding to points III, IV and VI in Fig. 3(**D**) of the main text.

For the pump power corresponding to point IV (V) in Fig. 3, we find that the condensate is a single mode (multi-mode) for all a values. At the critical point (i.e. the pump power corresponding to point III), we observed that mode switching only happens for $a = 1.1, 1.17$ μm, while for the small grid spacing ($a = 0.8$ μm and $a = 1.02$ μm), and for the larger grid spacing ($a = 1.55$ μm), we observed a single-mode condensate in each stochastic realization.

For the parameters considered in our work, the TWA condition reads $a \gg (g_{LP}/\gamma_{LP})^{1/2} = 0.85$ μm. $a = 0.8$ and $a = 1.02$ can therefore be excluded. At large grid spacing (i.e. $a \geq 1.55$ μm), the spatial discretization becomes too coarse for the typical scales of the system. Due to a very small high-momentum cut-off ($k_{max} = \pi/a$), momenta contributions that are still significant in the system are suppressed.

VI. QUANTUM FLUCTUATIONS VS. CLASSICAL NOISE

Let us first define what we mean by quantum fluctuations. The definition of quantum noise which we use in this study is the same as in the seminal book by Crispin Gardiner and Peter Zoller [59]. Fluctuations in quantum system arising from interactions with the external world leading to either drive or dissipation are defined as quantum noise. In contrast, fluctuations caused by finite temperature are referred to as classical noise. In the

truncated Wigner approximation (TWA), quantum fluctuations of the open system manifest themselves as an additive white noise in space and time present at every time step of the dynamics. This arises from the 2nd order derivative term of the Fokker-Planck (FP) equations from where TWA is derived. It has to be mentioned that TWA has also been used, especially in the context of cold atoms, to describe finite temperatures [60]. But, as it can be found in literature, in a closed system, the 2nd order term is missing in FP equations, and so there is no additive dynamical noise. Instead, the finite temperature fluctuations are encoded in the initial conditions. The dynamical equations remain mean-field and the thermal fluctuations are incorporated in the random/noisy initial conditions, where the observables are computed by averaging over different initial conditions.

Since our system is open (driven/dissipative) and weakly interacting, it is not in thermal equilibrium and the temperature is not defined. If we were to encode some temperature in the initial condition, as in cold atoms, this would have no effect on the steady state as due to continues drive and dissipation (i.e. noise at every time step), the influence of any initial conditions will be washed out. Our steady state is initial condition independent.

Based on timescales, we have identified two realistically relevant processes that can induce mode hopping; the quantum noise acting on photons due to their finite lifetime in the cavity, and the potential variations in the pump power (classical noise). Note that the real-valued noise corresponding to spatio-temporal variations in the pump gives fluctuations in the density only. There are no fluctuations in the phase of the condensate. What we can say for certain is that we need fluctuations in the phase to explain the experimental results. Fluctuations in density alone are not sufficient. That is why we believe that fluctuations in pump power are not the cause of the effects described here. There potentially could be another source of noise which gives phase fluctuations (external to polariton population) such as phonons or high energy excitons. However, we show in the next sections that interactions with phonons or high energy excitons are orders of magnitude weaker than the dissipation coming from the finite cavity photon lifetime.

A. Interaction with phonons

Modeling thermal fluctuations within the G-P model is a difficult task. To show that polariton-phonon interaction is weak, we take a different approach to estimate the polariton-phonon interaction, namely a quantum Boltzmann equation. To simulate the dynamics of the polaritons, we make use of the semiclassical Boltzmann equation, which reads:

$$\frac{\partial n_{\vec{k}}}{\partial t} = P_{\vec{k}}(t) - \frac{n_{\vec{k}}}{\tau_{\vec{k}}} + \sum_{\vec{k}'} W^{(i)}_{\vec{k}'\to\vec{k}}(t) - \sum_{\vec{k}'} W^{(i)}_{\vec{k}\to\vec{k}'}(t). \quad \text{(S8)}$$

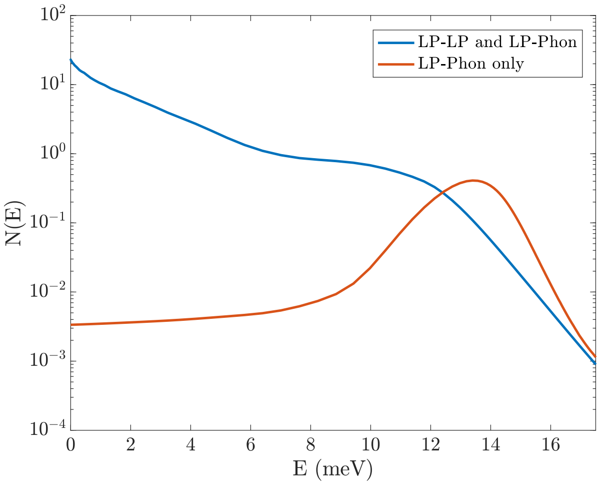

Figure S7: **Steady state occupation.** The occupation of the lower polaritons as a function of their energy obtained by solving Eq. S8 numerically. Red: when only the polariton-phonon interaction is considered. Blue: when both the lower polariton-phonon and polariton-polariton interactions are considered.

.

Here $n_{\vec{k}}$ is the occupation number, $\tau_{\vec{k}}$ is the characteristic lifetime, $P_{\vec{k}}$ is the pumping term. The $W^{(i)}$ are the interaction terms for particle-particle collisions. The laser generation is modeled by using a time-independent pump term P that is longer than the total simulated time (i.e. c.w pumping). Since the laser generation is non-resonant with energy much greater than the polariton energies, we assumed that the free electrons and holes created in the pump process lead each polariton state being pumped with equal probability. We solve Eq. (S8) numerically by including the polariton-phonon interaction and polariton-polariton interaction to find $n_{\vec{k}}$. The updated $n_{\vec{k}}(t)$ is then used to find the new $W_{\vec{k}\to\vec{k}'}(t)$ until a steady-state distribution is reached. The interaction terms for the polariton-polariton and polariton-phonon interactions are given in Ref [61]. The occupation number of the polartions obtained from Eq. (S8) as a function of their energy is shown in Fig. S7.

Figure S7 shows several important aspects about exciton-polaritons in general. First, the three regions of the energy distribution have very different properties. At high energy, which corresponds to the excitonic range of the spectrum, there is a thermal tail which fits a Maxwell-Boltzmann distribution since these high energy excitons interact very efficiently with phonons. The temperature of this distribution is very close to the lattice temperature. However, at low energy, which corresponds to the polaritonic region, the polaritons have a nearly flat distribution when only the polariton-phonon interaction is included and therefore do not have a well defined temperature since they interact very weakly with the lattice. For this reason, we believe that the dissipation coming

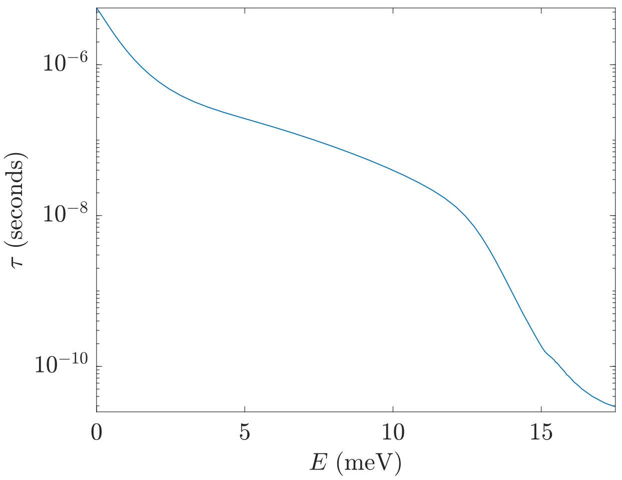

Figure S8: **Polariton-phonon scattering time.** The scattering time of polaritons with phonons as a function of the polariton energy calculated from Eq. S9

.

from the finite cavity photon lifetime is orders of magnitude larger than anything connected with interactions with phonons. When the polariton-polariton interaction is included, the polaritons have a nearly thermal distribution with a temperature well above the lattice temperature. What we find is that polaritons thermalize with each other and not with the lattice. This is consistent with Quantum Boltzmann solutions previously reported in the literature [61].

To be more concrete about the strength of this polariton-phonon interaction, we calculate the polariton-phonon out-scattering rate per particle in a momentum state k (Ref. [62], Chapter 4):

$$\frac{1}{\tau_{\vec{k}}} = \frac{1}{n_{\vec{k}}}\frac{dn_{\vec{k}}}{dt} = \frac{2\pi}{\hbar}\sum_{\vec{k}_1,\vec{q}_z}|M(\vec{k},\vec{q})|^2\left[n_{\vec{q}}^{\text{phon}} + \frac{1}{2} \pm \frac{1}{2}\right] \times \delta\left(E_{\text{LP}}(\vec{k}_1) - E_{\text{LP}}(\vec{k}) \pm \hbar\nu\vec{q}\right), \tag{S9}$$

where $\pm$ correpsond to phonon emission (+) and phonon absorption (−). Here $n_{\vec{k}}$ is the occupation number of the lower polariton and $n_{\vec{q}}^{\text{phon}}$ is the occupation number of the phonons, which we assume are in thermal equilibrium with $n_{\vec{q}}^{\text{phon}}$ given by the Planck distribution $n_{\vec{q}}^{\text{phon}} = 1/\left(e^{\hbar\omega_q/k_BT} - 1\right)$. The polariton-phonon interaction is based on hydrostatic deformation potential, which takes the form [63],

$$M(\vec{k},\vec{q}) = iX_kX_{k'}\sqrt{\frac{\hbar\left(q_\parallel^2 + q_z^2\right)^{1/2}}{2\rho V u}} \times \left[a_e I_e^\parallel\left((|\vec{q}|)I_e^\perp(q_z) - a_h I_h^\parallel(|\vec{q}|)I_h^\perp(q_z)\right)\right], \tag{S10}$$

where V, ρ, u are volume, density and longitudinal sound velocity respectively. X_k is the Hopfield coefficient and a_e and a_h are the deformation coefficients of the conduction and valence band for GaAs respectively. $I_{\text{e(h)}}^{\perp(\parallel)}$ are the overlap integrals between the exciton and phonon mode. The scattering time is plotted in Fig. S8. The scattering time in the polaritonic region is of the order of 10^{-6} seconds, which is negligible compared to the cavity photon decay, which is of the order of a few hundred picoseconds. The scattering time with phonons is four orders of magnitude weaker than the cavity photon decay. In the excitonic region, the scattering time is of the order of tens of picoseconds, which is why they can efficiently thermalize with phonons.

B. High energy excitons

In the experimental setup, the high energy excitons stay mostly near vicinity of the pump spot since they are 10^4 heavier than the polaritons. The diffusion length of these high energy excitons is typically $\leqslant 1$ μm while the diameter of the annular trap is 45 μm. We therefore expect that the density of these high energy excitons inside the trap where the condensate is forming is nearly zero and therfore, the effect of these high energy excitons on the polaritons inside the trap is negligible. The bottleneck excitons have some polaritonic character and do not exhibit the nearly stationary behavior [64]; this poses an interesting question, however, modeling such excitons is difficult and beyond the scope of this work.

REFERENCES AND NOTES

1. M. White, D. Scott, J. Silk, Anisotropies in the cosmic microwave background. *Annu. Rev. Astron. Astrophys.* **32**, 319–370 (1994).
2. H. B. Casimir, On the attraction between two perfectly conducting plates. *Proc. Kon. Ned. Akad. Wetensch. Proc.* **51**, 793–795 (1948).
3. A. Gambassi, The casimir effect: From quantum to critical fluctuations, (IOP Publishing, 2009), vol. 161 p. 012037.
4. S. Chakravarty, B. I. Halperin, D. R. Nelson, Twodimensional quantum heisenberg antiferromagnet at low temperatures. *Phys. Rev. B Condens. Matter.* **39**, 2344–2371 (1989).
5. C. Rüegg, B. Normand, M. Matsumoto, A. Furrer, D. F. McMorrow, K. W. Krämer, H.-U. Güdel, S. N. Gvasaliya, H. Mutka, M. Boehm, Quantum magnets under pressure: controlling elementary excitations in tlcucl 3. *Phys. Rev. Lett.* **100**, 205701 (2008).
6. A. Schröder, G. Aeppli, R. Coldea, M. Adams, O. Stockert, H. Löhneysen, E. Bucher, R. Ramazashvili, P. Coleman, Onset of antiferromagnetism in heavyfermion metals. *Nature* **407**, 351–355 (2000).
7. L. Chomaz, S. Baier, D. Petter, M. Mark, F. Wächtler, L. Santos, F. Ferlaino, Quantum-fluctuation-driven crossover from a dilute bose-einstein condensate to a macrodroplet in a dipolar quantum fluid. *Phys. Rev. X* **6**, 041039 (2016).
8. S. Papp, J. Pino, R. Wild, S. Ronen, C. E. Wieman, D. S. Jin, E. A. Cornell, Bragg spectroscopy of a strongly Interacting ^{85}Rb Bose-Einstein condensate. *Phys. Rev. Lett.* **101**, 135301 (2008).
9. M. Das, J. R. Green, Critical fluctuations and slowing down of chaos. *Nat. Commun.* **10**, 2155 (2019).
10. S. Giorgini, L. P. Pitaevskii, S. Stringari, Anomalous fluctuations of the condensate in interacting bose gases. *Phys. Rev. Lett.* **80**, 5040–5043 (1998).
11. F. Meier, W. Zwerger, Anomalous condensate fluctuations in strongly interacting superfluids. *Phys. Rev. A* **60**, 5133–5135 (1999).
12. M. A. Kristensen, M. B. Christensen, M. Gajdacz, M. Iglicki, K. Pawłowski, C. Klempt, J. F. Sherson, K. Rzążewski, A. J. Hilliard, J. J. Arlt, Observation of atom number fluctuations in a bose-einstein condensate. *Phys. Rev. Lett.* **122**, 163601 (2019).
13. J. Schmitt, T. Damm, D. Dung, F. Vewinger, J. Klaers, M. Weitz, Observation of grand-canonical number statistics in a photon bose-einstein condensate. *Phys. Rev. Lett.* **112**, 030401 (2014).
14. F. E. Ozturk, T. Lappe, G. Hellmann, J. Schmitt, J. Klaers, F. Vewinger, J. Kroha, M. Weitz, Fluctuation dynamics of an open photon bose-einstein condensate. *Phys. Rev. A* **100**, 043803 (2019).

15. J. Kasprzak, M. Richard, S. Kundermann, A. Baas, P. Jeambrun, J. M. J. Keeling, F. Marchetti, M. Szymańska, R. André, J. Staehli, V. Savona, P. B. Littlewood, B. Deveaud, L. S. Dang, Bose–einstein condensation of exciton polaritons. *Nature* **443**, 409–414 (2006).
16. R. Balili, V. Hartwell, D. Snoke, L. Pfeiffer, K. West, Bose-einstein condensation of microcavity polaritons in a trap. *Science* **316**, 1007–1010 (2007).
17. A. Amo, J. Lefrère, S. Pigeon, C. Adrados, C. Ciuti, I. Carusotto, R. Houdré, E. Giacobino, A. Bramati, Superfluidity of polaritons in semiconductor microcavities. *Nat. Phys.* **5**, 805–810 (2009).
18. K. G. Lagoudakis, M. Wouters, M. Richard, A. Baas, I. Carusotto, R. André, L. S. Dang, B. DeveaudPlédran, Quantized vortices in an exciton–polariton condensate. *Nat. Phys.* **4**, 706–710 (2008).
19. K. Lagoudakis, , Observation of Half-Quantum Vortices in an Exciton-Polariton Condensate. *Science* **326**, 974–976 (2009).
20. D. Sanvitto, F. Marchetti, M. Szymańska, G. Tosi, M. Baudisch, F. P. Laussy, D. Krizhanovskii, M. Skolnick, L. Marrucci, A. Lemaitre, J. Bloch, C. Tejedor, L. Viña, Persistent currents and quantized vortices in a polariton superfluid. *Nat. Phys.* **6**, 527–533 (2010).
21. G. Nardin, G. Grosso, Y. Léger, B. Piétka, F. MorierGenoud, B. Deveaud-Plédran, Hydrodynamic nucleation of quantized vortex pairs in a polariton quantum fluid. *Nat. Phys.* **7**, 635–641 (2011).
22. G. Tosi, G. Christmann, N. Berloff, P. Tsotsis, T. Gao, Z. Hatzopoulos, P. Savvidis, J. Baumberg, Geometrically locked vortex lattices in semiconductor quantum fluids. *Nat. Commun.* **3**, 1 (2012).
23. G. Liu, D. W. Snoke, A. Daley, L. N. Pfeiffer, K. West, A new type of half-quantum circulation in a macroscopic polariton spinor ring condensate. *Proc. Natl. Acad. Sci. U.S.A.* **112**, 2676–2681 (2015).
24. C. Degen, I. Fischer, W. Elsäßer, Transverse modes in oxide confined vcsels: Influence of pump profile, spatial hole burning, and thermal effects. *Opt. Express* **5**, 38–47 (1999).
25. B. Zhang, D. W. Snoke, A. P. Heberle, Towards the transverse mode-locking of oxide-confined vcsels. *Opt. Commun.* **285**, 4117–4119 (2012).
26. Q. Deng, H. Deng, D. Deppe, Radiation fields from whispering-gallery modes of oxide-confined vertical-cavity surface-emitting lasers. *Opt. Lett.* **22**, 463–465 (1997).
27. S. Pereira, M. Willemsen, M. Van Exter, J. Woerdman, Pinning of daisy modes in optically pumped vertical-cavity surface-emitting lasers. *Appl. Phys. Lett.* **73**, 2239–2241 (1998).
28. M. Steger, G. Liu, B. Nelsen, C. Gautham, D. W. Snoke, R. Balili, L. Pfeiffer, K. West, Long-range ballistic motion and coherent flow of long-lifetime polaritons. *Phys. Rev. B* **88**, 235314 (2013).

29. M. Steger, C. Gautham, D. W. Snoke, L. Pfeiffer, K. West, Slow reflection and two-photon generation of microcavity exciton–polaritons. *Optica* **2**, 1–5 (2015).
30. M. Steger, C. Gautham, B. Nelsen, D. Snoke, L. Pfeiffer, K. West, Single-wavelength, all-optical switching based on exciton-polaritons. *Appl. Phys. Lett.* **101**, 131104 (2012).
31. Y. Sun, P. Wen, Y. Yoon, G. Liu, M. Steger, L. N. Pfeiffer, K. West, D. W. Snoke, K. A. Nelson, Boseeinstein condensation of long-lifetime polaritons in thermal equilibrium. *Phys. Rev. Lett.* **118**, 016602 (2017).
32. D. Caputo, D. Ballarini, G. Dagvadorj, C. Sánchez Muñoz, M. De Giorgi, L. Dominici, K. West, L. N. Pfeiffer, G. Gigli, F. P. Laussy, M. H. Szymańska, D. Sanvitto, Topological order and thermal equilibrium in polariton condensates. *Nat. Mater.* **17**, 145–151 (2018).
33. Y. Sun, Y. Yoon, S. Khan, L. Ge, M. Steger, L. N. Pfeiffer, K. West, H. E. Türeci, D. W. Snoke, K. A. Nelson, Stable switching among high-order modes in polariton condensates. *Phys. Rev. B* **97**, 045303 (2018).
34. A. Dreismann, P. Cristofolini, R. Balili, G. Christmann, F. Pinsker, N. G. Berloff, Z. Hatzopoulos, P. G. Savvidis, J. J. Baumberg, Coupled counterrotating polariton condensates in optically defined annular potentials. *Proc. Natl. Acad. Sci. U.S.A.* **111**, 8770–8775 (2014).
35. A. Askitopoulos, T. Liew, H. Ohadi, Z. Hatzopoulos, P. Savvidis, P. Lagoudakis, Robust platform for engineering pure-quantum-state transitions in polariton condensates. *Phys. Rev. B* **92**, 035305 (2015).
36. F. Manni, K. G. Lagoudakis, T. C. H. Liew, R. André, B. Deveaud-Plédran, Spontaneous pattern formation in a polariton condensate. *Phys. Rev. Lett.* **107**, 106401 (2011).
37. P. Cristofolini, A. Dreismann, G. Christmann, G. Franchetti, N. Berloff, P. Tsotsis, Z. Hatzopoulos, P. Savvidis, J. Baumberg, Optical superfluid phase transitions and trapping of polariton condensates. *Phys. Rev. Lett.* **110**, 186403 (2013).
38. C. Ouellet-Plamondon, G. Sallen, F. Morier-Genoud, D. Oberli, M. Portella-Oberli, B. Deveaud, Spatial multistability induced by cross interactions of confined polariton modes. *Phys. Rev. B* **93**, 085313 (2016).
39. A. Baas, J. P. Karr, H. Eleuch, E. Giacobino, Optical bistability in semiconductor microcavities. *Phys. Rev. A* **69**, 023809 (2004).
40. M. Furman, A. Opala, M. Król, K. Tyszka, R. Mirek, M. Muszyński, B. Seredyński, W. Pacuski, J. Szczytko, M. Matuszewski, B. Piętka, Inverted optical bistability and optical limiting in coherently driven exciton–polaritons, *APL Photonics* **8**, 046105 (2023).

41. M. Ohtsu, Y. Otsuka, Y. Teramachi, Precise measurements and computer simulations of mode-hopping phenomena in semiconductor lasers. *Appl. Phys. Lett.* **46**, 108–110 (1985).
42. M. Ohtsu, Y. Teramachi, Y. Otsuka, A. Osaki, Analyses of mode-hopping phenomena in an algaas laser. *IEEE J. Quantum Electron.* **22**, 535–543 (1986).
43. M. Ohtsu, Y. Teramachi, Analyses of mode partition and mode hopping in semiconductor lasers. *IEEE J. Quantum Electron.* **25**, 31–38 (1989).
44. I. Carusotto, C. Ciuti, Quantum fluids of light. *Rev. Mod. Phys.* **85**, 299–366 (2013).
45. E. Estrecho, T. Gao, N. Bobrovska, M. D. Fraser, M. Steger, L. Pfeiffer, K. West, T. C. H. Liew, M. Matuszewski, D. W. Snoke, Single-shot condensation of exciton polaritons and the hole burning effect. *Nat. Commun.* **9**, 2944 (2018).
46. Similar to the real space images, the angle-resolved images are a sum of 42 laser pulses, each time integrated for 100 ms.
47. N. Bobrovska, M. Matuszewski, Adiabatic approximation and fluctuations in exciton-polariton condensates. *Phys. Rev. B* **92**, 035311 (2015).
48. A. Opala, M. Pieczarka, M. Matuszewski, Theory of relaxation oscillations in exciton-polariton condensates. *Phys. Rev. B* **98**, 195312 (2018).
49. M. De Giorgi, D. Ballarini, P. Cazzato, G. Deligeorgis, S. I. Tsintzos, Z. Hatzopoulos, P. G. Savvidis, G. Gigli, F. P. Laussy, D. Sanvitto, Relaxation oscillations in the formation of a polariton condensate. *Phys. Rev. Lett.* **112**, 113602 (2014).
50. F. Baboux, D. D. Bernardis, V. Goblot, V. N. Gladilin, C. Gomez, E. Galopin, L. L. Gratiet, A. Lemaître, I. Sagnes, I. Carusotto, M. Wouters, A. Amo, J. Bloch, Unstable and stable regimes of polariton condensation. *Optica* **5**, 1163 (2018).
51. A. V. Nalitov, H. Sigurdsson, S. Morina, Y. S. Krivosenko, I. V. Iorsh, Y. G. Rubo, A. V. Kavokin, I. A. Shelykh, Optically trapped polariton condensates as semiclassical time crystals. *Phys. Rev. A* **99**, 033830 (2019).
52. N. Bobrovska, M. Matuszewski, K. S. Daskalakis, S. A. Maier, S. Kéna-Cohen, Dynamical instability of a nonequilibrium exciton-polariton condensate. *ACS Photonics* **5**, 111–118 (2018).
53. C. Whittaker, B. Dzurnak, O. Egorov, G. Buonaiuto, P. Walker, E. Cancellieri, D. Whittaker, E. Clarke, S. Gavrilov, M. Skolnick, D. N. Krizhanovskii, Polariton pattern formation and photon statistics of the associated emission. *Phys. Rev. X* **7**, 031033 (2017).

54. H. Deng, H. Haug, Y. Yamamoto, Exciton-polariton bose-einstein condensation. *Rev. Mod. Phys.* **82**, 1489–1537 (2010).
55. P. Comaron, I. Carusotto, M. H. Szymańska, N. P. Proukakis, Non-equilibrium Berezinskii-Kosterlitz-Thouless transition in driven-dissipative condensates[(a)]. *EPL* **133**, 17002 (2021).
56. N. Bobrovska, E. A. Ostrovskaya, M. Matuszewski, Stability and spatial coherence of nonresonantly pumped exciton-polariton condensates. *Phys. Rev. B* **90**, 205304 (2014).
57. D. Snoke, V. Hartwell, J. Beaumariage, S. Mukherjee, Y. Yoon, D. Myers, M. Steger, Z. Sun, K. Nelson, L. Pfeiffer, Reanalysis of experimental determinations of polariton-polariton interactions in microcavities. *Phys. Rev. B* **107**, 165302 (2023).
58. P. Mietki, Exciton-Polariton Condensates with Internal Degrees of Freedom, Ph.D. thesis, Institute of Physics, Polish Academy of Science, Division of the Theoretical Physics (2021).
59. C. Gardiner and P. Zoller, *Quantum noise: A handbook of Markovian and non-Markovian quantum stochastic methods with applications to quantum optics* (Springer Science & Business Media, 2004).
60. N. P. Proukakis, B. Jackson, Finite-temperature models of bose–einstein condensation. *J. Phys. B: At. Mol. Opt. Phys.* **41**, 203002 (2008).
61. V. Hartwell, D. Snoke, Numerical simulations of the polariton kinetic energy distribution in gaas quantum-well microcavity structures. *Phys. Rev. B* **82**, 075307 (2010).
62. D. W. Snoke, *Solid state physics: Essential concepts* (Cambridge Univ. Press, 2020).
63. C. Piermarocchi, F. Tassone, V. Savona, A. Quattropani, P. Schwendimann, Nonequilibrium dynamics of free quantum-well excitons in time-resolved photoluminescence. *Phys. Rev. B* **53**, 15834–15841 (1996).
64. D. Myers, S. Mukherjee, J. Beaumariage, D. Snoke, M. Steger, L. Pfeiffer, K. West, Polariton-enhanced exciton transport. *Phys. Rev. B* **98**, 235302 (2018).
